# Fuzzy *C*_*e*_-*I*(ec, eo) and Fuzzy Completely *C*_*e*_-*I*(rc, eo) Functions via Fuzzy e-Open Sets

**DOI:** 10.1155/2016/2587875

**Published:** 2016-03-08

**Authors:** V. Seenivasan, K. Kamala

**Affiliations:** Department of Mathematics, University College of Engineering Panruti (A Constituent College of Anna University, Chennai), Panruti, Tamil Nadu 607106, India

## Abstract

We introduced the notions of fuzzy *C*_*e*_-*I*(ec, eo) functions and fuzzy completely *C*_*e*_-*I*(rc, eo) functions via fuzzy e-open sets. Some properties and several characterization of these types of functions are investigated.

## 1. Introduction

With the introduction of fuzzy sets by Zadeh [1] and fuzzy topology by Chang [2], the theory of fuzzy topological spaces was subsequently developed by several fuzzy topologist based on the concepts of general topology. In 2014, the concept of fuzzy e-open sets and fuzzy e-continuity and separations axioms and their properties were defined by Seenivasan and Kamala [3]. In this paper, we introduce the notion of fuzzy *C*_*e*_-*I*(ec, eo) functions, fuzzy *C*_*e*_-continuous, fuzzy completely *C*_*e*_-*I*(rc, eo) functions, and fuzzy e-kernel via fuzzy e-open sets and studied their properties and several characterizations of these types of functions are investigated. In this paper, we denote fuzzy e-open, fuzzy e-closed, and fuzzy regular closed as, eo, ec, and rc, respectively.

## 2. Preliminaries

Throughout this paper, (*X*, *τ*) and (*Y*, *σ*) (or simply *X* and *Y*) represent nonempty fuzzy topological spaces on which no separation axioms are assumed, unless otherwise mentioned.

Let *μ* be any fuzzy set of *X*. The fuzzy closure of *μ*, fuzzy interior of *μ*, fuzzy *δ*-closure of *μ*, and the fuzzy *δ*-interior of *μ* are denoted by cl(*μ*), int(*μ*), cl_*δ*_(*μ*), and int_*δ*_(*μ*), respectively. A fuzzy set *μ* of *X* is called fuzzy regular open [4] (resp., fuzzy regular closed) if *μ* = int(cl(*μ*)) (resp., *μ* = cl(int(*μ*))).

The fuzzy *δ*-interior of fuzzy set *μ* of *X* is the union of all fuzzy regular open sets contained in *μ*. A fuzzy set *μ* is called fuzzy *δ*-open [5] if *μ* = int_*δ*_(*μ*). The complement of fuzzy *δ*-open set is called fuzzy *δ*-closed (i.e, *μ* = cl_*δ*_(*μ*)). A fuzzy set *μ* of *X* is called fuzzy *δ*-preopen [6] (resp., fuzzy *δ*-semi open [7]) if *μ* ≤ int(cl_*δ*_(*μ*)) (resp., *μ* ≤ cl(int_*δ*_(*μ*))). The complement of a fuzzy *δ*-preopen set (resp., fuzzy *δ*-semiopen set) is called fuzzy *δ*-preclosed (resp., fuzzy *δ*-semiclosed).

**Definition 1 .**

A fuzzy set *μ* of a fuzzy topological space *X* is called fuzzy e-open [3] if *μ* ≤ cl(int_*δ*_ *μ*)∨int(cl_*δ*_ *μ*). Fuzzy e-closed if *μ* ≥ cl(int_*δ*_ *μ*)∧int(cl_*δ*_ *μ*).

The intersection of all fuzzy e-closed sets containing *μ* is called fuzzy e-closure of *μ* and is denoted by fe-cl(*μ*) and the union of all fuzzy e-open sets contained in *μ* is called fuzzy e-interior of *μ* and is denoted by fe-int(*μ*).

**Definition 2 .**

A mapping *f* : *X* → *Y* is said to be fuzzy e^*∗*^-open [8] if the image of every fuzzy e-open set in *X* is fuzzy e-open set in *Y*.

**Definition 3 .**

A function *f* : *X* → *Y* is called fuzzy e-irresolute [3]. *f* ^−1^(*λ*) is fuzzy e-open in *X* for every fuzzy e-open set *λ* of *Y*.

**Definition 4 .**

A fuzzy set *μ* is quasicoincident [9] with a fuzzy set *λ* denoted by *μqλ* iff there exist *x* ∈ *X* such that *μ*(*x*) + *λ*(*x*) > 1. If *μ* and *λ* are not quasicoincident, then we write $\mu \bar{q}\lambda$ and $\mu \leq \lambda \Leftrightarrow \mu \bar{q}1-\lambda .$

**Definition 5 .**

A fuzzy point *x*_*p*_ is quasicoincident [9] with a fuzzy set *λ* denoted by *x*_*p*_ *qλ* iff there exist *x* ∈ *X* such that *p* + *λ*(*x*) > 1.

**Definition 6 .**

A fuzzy topological space (*X*, *τ*) is said to be fuzzy *e*-*T*_1_ [3] if for each pair of distinct points *x* and *y* of *X* there exist fuzzy e-open sets *μ*_1_and *μ*_2_ such that *x* ∈ *μ*_1_ and *y* ∈ *μ*_2_ and *x* ∉ *μ*_2_ and *y* ∉ *μ*_1_.

**Definition 7 .**

A fuzzy topological space (*X*, *τ*) is said to be fuzzy *e*-*T*_2_ [3] if for each pair of distinct points *x* and *y* of *X* there exists disjoint fuzzy e-open sets *η* and *ρ* such that *x* ∈ *η* and *y* ∈ *ρ*.

**Definition 8 .**

A fuzzy topological space *X* is said to be fuzzy weakly Hausdorff [10] if each element of *X* is an intersection of fuzzy regular closed sets.

**Definition 9 .**

A fuzzy topological space *X* is said to be fuzzy e-normal [3] if for every two disjoint fuzzy closed sets *η* and *ρ* of *X* there exist two disjoint fuzzy e-open sets *μ* and *λ* such that *η* ≤ *μ* and *ρ* ≤ *λ* and *μ*∧*λ* = 0.

**Definition 10 .**

A fuzzy topological space *X* is said to be fuzzy strongly normal [10] if for every two disjoint fuzzy closed sets *η* and *ρ* of *X* there exist two disjoint fuzzy open sets *μ* and *λ* such that *η* ≤ *μ* and *ρ* ≤ *λ*.

**Definition 11 .**

A fuzzy topological space *X* is said to be fuzzy Urysohn [11] if for every distinct points *x* and *y* in *X* there exist fuzzy open sets *μ* and *λ* in *X* such that *x* ∈ *μ* and *y* ∈ *λ* and cl(*μ*)∧cl(*λ*) = 0.

**Definition 12 .**

A space (*X*, *τ*) is called fuzzy S-closed [2] (resp., fuzzy e-compact [3]) if every fuzzy regular closed (resp., fuzzy e-open) cover of *X* has a finite subcover.

**Definition 13 .**

A function *f* : *X* → *Y* is called fuzzy completely continuous [12] if *f* ^−1^(*λ*) is fuzzy regular open in *X* for every fuzzy open set *λ* of *Y*.

**Definition 14 .**

A fuzzy filter base *ξ* is said to be fuzzy rc-convergent [10] to a fuzzy point *x*_*ε*_ in *X* if for any fuzzy regular closed set *η* in *X* containing *x*_*ε*_ there exists a fuzzy set *ρ* ∈ *ξ* such that *ρ* ≤ *η*.

**Definition 15 .**

A collection of fuzzy subsets Δ of a fuzzy topological spaces *X* is said to form fuzzy filterbases [13] iff for every finite collection {*λ*_*α*_ : *α* = 1,2,…, *n*}, ⋀_*α*=1_^*n*^ *λ*_*α*_ ≠ 0_*X*_.

## 3. Fuzzy *C*_*e*_-*I*(ec, eo) Functions

In this section, the notion of fuzzy *C*_*e*_-*I*(ec, eo) functions is introduced and some characteristics and properties are studied.

**Definition 16 .**

A mapping *φ* : (*X*, *τ*)→(*Y*, *σ*) is called fuzzy *C*_*e*_-*I*(ec, eo) if the inverse image of every fuzzy e-open set of *Y* is fuzzy e-closed in *X*.

**Remark 17 .**

The concepts of fuzzy *C*_*e*_-*I*(ec, eo) and fuzzy e-irresolute are independent notions as illustrated in the following example.

**Example 18 .**

Let *X* = {*a*, *b*, *c*} and *Y* = {*x*, *y*, *z*} and the fuzzy sets *μ*_1_, *μ*_2_, *υ* be defined as follows:

(1) μ1a=0.1,μ2a=0.7,υx=0.2,μ1b=0.6,μ2b=0.5,υy=0.2,μ1c=0.4,μ2c=0.6,υz=0.4.

Let *τ* = {0,1, *μ*_1_, *μ*_2_, *μ*_1_∨*μ*_2_, *μ*_1_∧*μ*_2_} and *σ* = {0, *υ*, 1}. Then, the mapping *φ* : (*X*, *τ*)→(*Y*, *σ*) is defined by *φ*(*a*) = *x*, *φ*(*b*) = *y*, *φ*(*c*) = *z*. Then, *φ* is fuzzy *C*_*e*_-*I*(ec, eo) but not fuzzy e-irresolute.

**Example 19 .**

Let *X* = {*a*, *b*, *c*} and *Y* = {*x*, *y*, *z*} and the fuzzy sets *η*_1_, *η*_2_, *ρ* are defined as follows:

(2) η1a=0.4,η2a=0.3,ρx=0.4,η1b=0.7,η2b=0.5,ρy=0.5,η1c=0.8,η2c=0.2,ρz=1.

Let *τ* = {0,1, *η*_1_, *η*_2_} and *σ* = {0, *ρ*, 1}. Then, the mapping *φ* : (*X*, *τ*)→(*Y*, *σ*) is defined by *φ*(*a*) = *x*, *φ*(*b*) = *y*, *φ*(*c*) = *z*. Then, *φ* is fuzzy e-irresolute but not fuzzy *C*_*e*_-*I*(ec, eo).

**Definition 20 .**

A mapping *φ* : (*X*, *τ*)→(*Y*, *σ*) is called fuzzy *C*_*e*_-continuous if the inverse image of every fuzzy open set of *Y* is fuzzy e-closed in *X*.

**Remark 21 .**

Every fuzzy *C*_*e*_-*I*(ec, eo) function is fuzzy *C*_*e*_-continuous, but not conversely from the following example.

**Example 22 .**

Let *X* = {*a*, *b*, *c*} and *Y* = {*x*, *y*, *z*} and the fuzzy sets *μ*_1_, *μ*_2_, *ν*, *υ* are defined as follows:

(3) μ1a=0.6,μ2a=0.4,υa=0.8,νx=0.3,μ1b=0.5,μ2b=0.7,υb=0.5,νy=0.4,μ1c=0.3,μ2c=0.5,υc=0.3,νz=0.6.

Let *τ* = {0,1, *μ*_1_, *μ*_2_, *μ*_1_∨*μ*_2_, *μ*_1_∧*μ*_2_} and *σ* = {0, *ν*, 1}. Then, the mapping *φ* : (*X*, *τ*)→(*Y*, *σ*) is defined by *φ*(*a*) = *x*, *φ*(*b*) = *y*, and *φ*(*c*) = *z*. Then, *φ* is fuzzy *C*_*e*_-continuous but not fuzzy *C*_*e*_-*I*(ec, eo) as the fuzzy set *υ* is fuzzy e-open in *Y* but *φ* ^−1^(*υ*) is not fuzzy e-closed set in *X*.

**Theorem 23 .**

For a fuzzy function *φ* : *X* → *Y*, if *φ*(*x*_*ε*_)*qμ*, the inverse image of for every fuzzy e-closed set of *Y* is fuzzy e-open in *X* iff for any *x*_*ε*_ ∈ *X*; if *φ*(*x*_*ε*_)*qμ*, then *x*_*ε*_ *qfe*-int(*φ* ^−1^(*μ*)).

**Proof**

Let *μ* ≤ *Y* be a fuzzy e-closed set and *φ*(*x*_*ε*_)*qμ*. Then, *x*_*ε*_ *qφ* ^−1^(*μ*) and, by hypothesis, *φ* ^−1^(*μ*) = fe-int(*φ* ^−1^(*μ*)). We obtain, *x*_*ε*_ *q*fe-int(*φ* ^−1^(*μ*)). Converse can be shown easily.

**Theorem 24 .**

For a fuzzy function *φ* : *X* → *Y*, if *φ*(*x*_*ε*_)*qμ*, for any fuzzy e-closed set *μ* ≤ *Y* and for any *x*_*ε*_ ∈ *X*, *x*_*ε*_ *qfe*-int(*φ* ^−1^(*μ*)) iff there exists a fuzzy e-open set *δ* such that *x*_*ε*_ *qδ* and *φ*(*δ*) ≤ *μ*.

**Proof**

Let *μ* ≤ *Y* be any fuzzy e-closed set and let *φ*(*x*_*ε*_)*qμ*. Then, *x*_*ε*_ *q*fe-int(*φ* ^−1^(*μ*)). Take *δ* = fe-int(*φ* ^−1^(*μ*)) then *φ*(*δ*) = *φ*(fe-int(*φ* ^−1^(*μ*))) ≤ *φ*(*φ* ^−1^(*μ*)) ≤ *μ*, and *δ* is fuzzy e-open in *X* and *x*_*ε*_ *qδ*.

Conversely, let *μ* ≤ *Y* be any fuzzy e-closed set and let *φ*(*x*_*ε*_)*qμ*. By hypothesis, there exists fuzzy e-open set *δ* such that *x*_*ε*_ *qδ* and *φ*(*δ*) ≤ *μ*. This implies, *δ* ≤ *φ* ^−1^(*μ*) and then *x*_*ε*_ *q*fe-int(*φ* ^−1^(*μ*)).

**Theorem 25 .**

For a fuzzy function *φ* : *X* → *Y*, the following statements are equivalent:

(1) *f*  is fuzzy *C*_*e*_-*I*(*ec*, *eo*).
(2) For every fuzzy e- closed set *μ* in *Y*, *φ* ^−1^(*μ*) is fuzzy e-open in *X*.
(3) For every fuzzy open set *μ*, *φ* ^−1^(*fe*-int(*μ*)) is fuzzy e-closed.
(4) For every fuzzy closed set *η*, *φ* ^−1^(*fe*-cl⁡(*η*)) is fuzzy e-open.
(5) For each *x*_*ε*_ ∈ *X* and each fuzzy e-closed set *μ* in *Y* containing *φ*(*x*_*ε*_), there exists a fuzzy e-open set *ρ* in *X* containing *x*_*ε*_ such that *φ*(*ρ*) ≤ *μ*.
(6) For each *x*_*ε*_ ∈ *X* and each fuzzy e-open set *μ* in *Y* noncontaining *φ*(*x*_*ε*_), there exists a fuzzy e-closed set *ν* in *X* noncontaining *x*_*ε*_ such that *φ* ^−1^(*μ*) ≤ *ν*.

**Proof**

(1) ⇔ (2): let *ρ* be a fuzzy e-open set in *Y*. Then, 1_*Y*_ − *ρ* is fuzzy e-closed. By (2), *φ* ^−1^(1_*Y*_ − *ρ*) = 1_*X*_ − *φ* ^−1^(*ρ*) is fuzzy e-open. Thus, *φ* ^−1^(*ρ*) is fuzzy e-closed. Converse can be shown easily.

(1) ⇔ (3): let *μ* be a fuzzy open set. Since fe-int(*μ*) is fuzzy e-open, then by (1) it follows that *φ* ^−1^(fe-int(*μ*)) is fuzzy e-closed. The converse is easy to prove.

(2) ⇔ (4): let *η* be a fuzzy closed set. Since fe-cl(*η*) is fuzzy e-closed set, then by (2) it follows that *φ* ^−1^(fe-cl(*η*)) is fuzzy e-open. The converse is easy to prove.

(2) ⇔ (5): let *μ* be any fuzzy e-closed set in *Y* containing *φ*(*x*_*ε*_). By (2), *φ* ^−1^(*μ*) is fuzzy e-open set in *X* and *x*_*ε*_ ∈ *φ* ^−1^(*μ*). Take *ρ* = *φ* ^−1^(*μ*). Then, *φ*(*ρ*) ≤ *μ*. The converse can be shown easily.

(5) ⇔ (6): let *μ* be any fuzzy e-open set in *Y* noncontaining *φ*(*x*_*ε*_). Then, 1 − *μ* is a fuzzy e-closed set containing *φ*(*x*_*ε*_). By (5), there exists a fuzzy e-open set *ρ* in *X* containing *x*_*ε*_ such that *φ*(*ρ*) ≤ 1 − *μ*. Hence, *ρ* ≤ *φ* ^−1^(1 − *μ*) = 1 − *φ* ^−1^(*μ*) and *φ* ^−1^(*μ*) ≤ 1 − *ρ*. Take *ν* = 1 − *ρ*. We obtain that *ν* is a fuzzy e-closed set in *X* noncontaining *x*_*ε*_. The converse can be shown easily.

**Theorem 26 .**

Let *ϕ* : *X* → *Y* be a function and let *φ* : *X* → *X* × *Y* be the fuzzy graph function of *ϕ*, defined by *φ*(*x*_*ε*_) = (*x*_*ε*_, *ϕ*(*x*_*ε*_)) for every *x*_*ε*_ ∈ *X*. If *φ* is fuzzy *C*_*e*_-*I*(*ec*, *eo*), then *ϕ* is fuzzy *C*_*e*_-*I*(*ec*, *eo*).

**Proof**

Let *μ* be a fuzzy e-closed set in *Y*; then, 1_*X*_ × *μ* is a fuzzy e-closed set in *X* × *Y*. Since *φ* is fuzzy *C*_*e*_-*I*(ec, eo), then *ϕ* ^−1^(*μ*) = *φ* ^−1^(1_*X*_ × *μ*) is fuzzy e-open in *X*. Thus, *ϕ* is fuzzy *C*_*e*_-*I*(ec, eo).

**Theorem 27 .**

Let {*Y*_*λ*_ : *λ* ∈ Λ} be a family of product spaces. If a function *φ* : *X* → ∏*Y*_*λ*_ is fuzzy *C*_*e*_-*I*(*ec*, *eo*), then *P*_*λ*_∘*φ* : *X* → *Y*_*λ*_ is fuzzy *C*_*e*_-*I*(*ec*, *eo*) for each *λ* ∈ Λ where *P*_*λ*_ is the projection of ∏⁡*Y*_*λ*_ onto *Y*_*λ*_.

**Proof**

Let *δ* be any fuzzy e-open set in *Y*_*λ*_. Since *P*_*λ*_ is a fuzzy continuous and fuzzy open set, it is a fuzzy e-open set. Now *P*_*λ*_ : ∏*Y*_*λ*_ → *Y*_*λ*_, *P*_*λ*_^−1^(*δ*) is a fuzzy e-open in ∏*Y*_*λ*_. Therefore, *P*_*λ*_ is a fuzzy e-irresolute function. Now (*P*_*λ*_∘*φ*)^−1^(*δ*) = *φ* ^−1^(*P*_*λ*_^−1^(*δ*)), since *φ* is fuzzy *C*_*e*_-*I*(ec, eo). Hence *φ* ^−1^(*P*_*λ*_^−1^(*δ*)) is a fuzzy e-closed set, since *P*_*λ*_^−1^(*δ*) is a fuzzy e-open set. Hence, *P*_*λ*_∘*φ* is fuzzy *C*_*e*_-*I*(ec, eo).

**Theorem 28 .**

If the function *φ* : ∏*X*_*λ*_ → ∏*Y*_*λ*_ is fuzzy *C*_*e*_-*I*(*ec*, *eo*), then *φ*_*λ*_ : *X*_*λ*_ → *Y*_*λ*_ is fuzzy *C*_*e*_-*I*(*ec*, *eo*) for each *λ* ∈ Λ.

**Proof**

Let *λ*_0_ ∈ Λ be an arbitrary fixed index and let *υ*_*λ*_0__ be any fuzzy e-open set of *Y*_*λ*_0__; then, ∏*Y*_*μ*_ × *υ*_*λ*_0__ is fuzzy e-open in ∏*Y*_*λ*_, where *λ*_0_ ≠ *μ* ∈ Λ. Since *φ* is fuzzy *C*_*e*_-*I*(ec, eo) function, then *φ* ^−1^(∏*Y*_*μ*_ × *υ*_*λ*_0__) = ∏*X*_*μ*_ × *φ*_*λ*_0__^−1^(*υ*_*λ*_0__) is fuzzy e-closed in ∏*X*_*λ*_ and hence *φ*_*λ*_0__^−1^(*V*_*λ*_0__) is fuzzy e-closed in *X*_*λ*_0__. This implies *φ*_*λ*_0__ is fuzzy *C*_*e*_-*I*(ec, eo).

**Theorem 29 .**

If *φ* : *X* → *Y* is fuzzy *C*_*e*_-*I*(*ec*, *eo*) and *δ* is fuzzy closed set of *X*, then *φ*|_*δ*_ : *δ* → *Y* is fuzzy *C*_*e*_-*I*(*ec*, *eo*).

**Proof**

Let *λ* be a fuzzy e-open set of *Y*; then, (*φ*|_*δ*_)^−1^(*λ*) = *φ* ^−1^(*λ*)∧*δ*. Since *φ* ^−1^(*λ*) and *δ* are fuzzy closed, hence (*φ*|_*δ*_)^−1^(*λ*) is fuzzy e-closed in the relative topology of *δ*.

**Definition 30 .**

The intersection of all fuzzy e-open set *η* of a fuzzy topological space (*X*, *τ*) containing *μ* is called the fuzzy e-kernel of *μ* (briefly, fe-*K*_*μ*_), fe-*K*_*μ*_ = ⋀{*η* : *μ* ≤ *η*, *η*  is  fuzzy  e-open  set  of  *X*}.

The following properties hold for fuzzy sets *μ*, *λ* of *X*:

(1) *x* ∈ fe-*K*_*μ*_ iff *μ*∧*γ* ≠ 0 for any fuzzy e-closed set *γ* containing *x*.
(2) *μ* ≤ fe-*K*_*μ*_ and *μ* = fe-*K*_*μ*_ if *μ* is fuzzy e-open in *X*.
(3) *μ* ≤ *λ*; then, fe-*K*_*μ*_ ≤ fe-*K*_*λ*_.

**Theorem 31 .**

For a fuzzy function *φ* : *X* → *Y*, the following statements are equivalent:

(1) *φ* is fuzzy *C*_*e*_-*I*(*ec*, *eo*).
(2) *φ*(*fe*-cl(*μ*)) ≤ *fe*-*K*_*φ*(*μ*)_ for every fuzzy set *μ* of *X*.
(3) *fe*-cl⁡(*φ* ^−1^(*η*)) ≤ *φ* ^−1^(*fe*-*K*_*η*_) for every fuzzy set *η* of *Y*.

**Proof**

(1)⇒(2): let *μ* ≤ *X* and *y* ∉ fe-*K*_*φ*(*μ*)_. There exists a fuzzy e-closed set *γ* in *Y*, such that *y* ∈ *γ* and *φ*(*μ*)∧*γ* = 0. Therefore, *φ* ^−1^(*φ*(*μ*)∧*γ*) = 0. This implies that *μ*∧*φ* ^−1^(*γ*) = 0 and fe-cl(*μ*)∧*φ* ^−1^(*γ*) = 0. Thus, *φ*(fe-cl*μ*)∧*γ* = 0 and *y* ∉ *φ*(fe-cl*μ*). Hence, *φ*(fe-cl*φ*(*μ*)) ≤ fe-*K*_*φ*(*μ*)_.

(2)⇒(3): let *η* ≤ *Y*; then, *φ* ^−1^(*η*) ≤ *X*. By hypothesis, *φ*(fe-cl*φ* ^−1^(*η*)) ≤ fe-*K*_*φ*(*φ*^−1^(*η*))_ ≤ fe-*K*_*η*_. Hence, fe-cl(*φ* ^−1^(*η*)) ≤ *φ* ^−1^(fe-*K*_*η*_).

(3)⇒(1): let *η* be any fuzzy e-open set of *Y*; we have fe-cl(*φ* ^−1^(*η*)) ≤ *φ* ^−1^(fe-*K*_*η*_) = *φ* ^−1^(*η*), since *η* is fuzzy e-open and fe-cl(*φ* ^−1^(*η*)) = *φ* ^−1^(*η*). This implies that *φ* ^−1^(*η*) is fuzzy e-closed in *X*.

**Definition 32 .**

The fuzzy e-Frontier of a fuzzy set *γ* of a fuzzy topological space *X* is given by fe-Fr(*γ*) = fe-cl(*γ*)∧fe-cl(1_*X*_ − *γ*).

**Theorem 33 .**

The fuzzy point *x*_*ε*_ ∈ *X* such that *φ* : *X* → *Y* is not fuzzy *C*_*e*_-*I*(*ec*, *eo*) is exactly the union of fuzzy e-Frontier if the inverse image of the fuzzy e-closed set in *Y* contains *φ*(*x*_*ε*_).

**Proof**

Suppose that *φ* is not fuzzy *C*_*e*_-*I*(ec, eo) at the point *x*_*ε*_ ∈ *X*; then there exists a fuzzy e-closed set *γ* such that *φ*(*x*_*ε*_) ∈ *γ* and *φ*(*μ*)∧(1_*Y*_ − *γ*) ≠ 0 for all fuzzy e-open set *μ* such that *x*_*ε*_ ∈ *μ*. It follows that *μ*∧*φ* ^−1^(1_*Y*_ − *γ*) ≠ 0 and hence *x*_*ε*_ ∈ fe-cl*φ* ^−1^(1_*Y*_ − *γ*) = fe-cl(1_*X*_ − *φ* ^−1^(*γ*)). Thus, *x*_*ε*_ ∈ *φ* ^−1^(*γ*) ≤ fe-cl(*φ* ^−1^(*γ*)) and hence *x*_*ε*_ ∈ fe-Fr(*φ* ^−1^(*γ*)).

Conversely, suppose that *x*_*ε*_ ∈ fe-Fr(*φ* ^−1^(*γ*)), *γ* is fuzzy e-closed set of *Y* containing *φ*(*x*_*ε*_), and *φ* is fuzzy *C*_*e*_-*I*(ec, eo) at *x*_*ε*_ ∈ *X*. There exists fuzzy e-open set *μ* such that *x*_*ε*_ ∈ *μ* and *μ* ≤ *φ* ^−1^(*γ*). Thus, *x*_*ε*_ ∈ fe-int*φ* ^−1^(*γ*) and hence *x*_*ε*_ ∉ fe-Fr(*φ* ^−1^(*γ*)) for each fuzzy e-closed set *γ* of *Y* containing *φ*(*x*_*ε*_), a contradiction. Therefore, *φ* is not fuzzy *C*_*e*_-*I*(ec, eo).

**Theorem 34 .**

The following hold for functions *ϕ* : *X* → *Y* and *φ* : *Y* → *Z*:

(a) If *ϕ* : *X* → *Y* is fuzzy *C*_*e*_-*I*(*ec*, *eo*) and *φ* : *Y* → *Z* is fuzzy *C*_*e*_-continuous then *φ*∘*ϕ* : *X* → *Z* is fuzzy *C*_*e*_-continuous.
(b) If *ϕ* : *X* → *Y* is fuzzy *C*_*e*_-*I*(*ec*, *eo*) and *φ* : *Y* → *Z* is fuzzy e-irresolute then *φ*∘*ϕ* : *X* → *Z* is fuzzy *C*_*e*_-*I*(*ec*, *eo*).

**Theorem 35 .**

If *ϕ* : *X* → *Y* is a fuzzy e-irresolute surjective function and *φ* : *Y* → *Z* is a fuzzy function such that *φ*∘*ϕ* : *X* → *Z* is fuzzy *C*_*e*_-*I*(*ec*, *eo*), then *φ* is fuzzy *C*_*e*_-*I*(*ec*, *eo*).

**Proof**

Let *η* be any fuzzy e-closed set in *Z*. Since *φ*∘*ϕ* is fuzzy *C*_*e*_-*I*(ec, eo), (*φ*∘*ϕ*)^−1^(*η*) is fuzzy e-open in *X*. Therefore, *ϕ* ^−1^(*φ* ^−1^(*η*)) = (*φ*∘*ϕ*)^−1^(*η*) is fuzzy e-open in *X*. Since *ϕ* is fuzzy e-irresolute, surjection implies *ϕ*(*ϕ* ^−1^(*φ* ^−1^(*η*))) = *φ* ^−1^(*η*) is fuzzy e-open in *Y*. Thus, *φ* is fuzzy *C*_*e*_-*I*(ec, eo).

**Theorem 36 .**

If *ϕ* : *X* → *Y* is a fuzzy *e* ^*∗*^ − *open* surjective function and *φ* : *Y* → *Z* is a fuzzy function such that *φ*∘*ϕ* : *X* → *Z* is fuzzy *C*_*e*_-continuous, then *φ* is fuzzy *C*_*e*_-continuous.

**Proof**

Let *η* be any fuzzy closed set in *Z*. Since *φ*∘*ϕ* is fuzzy *C*_*e*_-continuous, (*φ*∘*ϕ*)^−1^(*η*) is fuzzy e-open in *X*. Therefore, *ϕ* ^−1^(*φ* ^−1^(*η*)) = (*φ*∘*ϕ*)^−1^(*η*) is fuzzy e-open in *X*. Since *ϕ* is fuzzy e^*∗*^-open, surjection implies *ϕ*(*ϕ* ^−1^(*φ* ^−1^(*η*))) = *φ* ^−1^(*η*) is fuzzy e-open in *Y*. Thus, *φ* is fuzzy *C*_*e*_-continuous.

## 4. Fuzzy Completely *C*_*e*_-*I*(rc, eo) Functions

In this section, the notion of fuzzy completely *C*_*e*_-*I*(rc, eo) functions is introduced and the relation between other functions is studied and further some structure preservation properties are investigated.

**Definition 37 .**

A mapping *φ* : (*X*, *τ*)→(*Y*, *σ*) is called fuzzy completely *C*_*e*_-*I*(rc, eo) if inverse image of every fuzzy e-open set in *Y* is fuzzy regular closed in *X*.

**Example 38 .**

Let *X* = {*x*, *y*, *z*} and the fuzzy sets *μ*_1_, *μ*_2_ are defined as follows:

(4) μ1x=0.4,μ2x=0.7,μ1y=0.6,μ2y=0.3,μ1z=0.1,μ2z=0.5.

Let *τ* = {0,1, *μ*_1_, *μ*_2_, *μ*_1_∨*μ*_2_, *μ*_1_∧*μ*_2_} and *σ* = {0, *μ*_1_, *μ*_1_∧*μ*_2_, 1}. Then, the mapping *φ* : (*X*, *τ*)→(*X*, *σ*) is defined by *φ*(*x*) = 1 − *x*. Then, *φ* is fuzzy completely *C*_*e*_-*I*(rc, eo).

**Remark 39 .**

Every fuzzy completely *C*_*e*_-*I*(rc, eo) function is fuzzy *C*_*e*_-*I*(ec, eo) and fuzzy *C*_*e*_-continuous, but the converse is not true, which can be seen in the following example.

**Example 40 .**

Let *X* = {*a*, *b*, *c*} and *Y* = {*x*, *y*, *z*} and the fuzzy sets *μ*_1_, *μ*_2_, and *ν* are defined as follows:

(5) μ1a=0.6,μ2a=0.3,νx=0.4,μ1b=0.5,μ2b=0.7,νy=0.3,μ1c=0.2,μ2c=0.8,νz=0.1.

Let *τ* = {0,1, *μ*_1_, *μ*_2_, *μ*_1_∨*μ*_2_, *μ*_1_∧*μ*_2_} and *σ* = {0, *ν*, 1}. Then, the mapping *φ* : (*X*, *τ*)→(*Y*, *σ*) is defined by *φ*(*a*) = *x*, *φ*(*b*) = *y*, *φ*(*c*) = *z*. Then, *φ* is fuzzy *C*_*e*_-continuous and also fuzzy *C*_*e*_-*I*(ec, eo) but not fuzzy completely *C*_*e*_-*I*(rc, eo) as the fuzzy set *υ* is fuzzy e-open in *Y* but *φ* ^−1^(*υ*) is not fuzzy regular closed set in *X*.

From the above examples, we have the following implications.


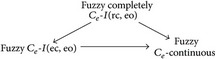


None of the these implications is reversible.

**Theorem 41 .**

For a fuzzy function *φ* : *X* → *Y*, if *φ*(*x*_*ε*_)*qμ*, the inverse image of every fuzzy e-closed set of *Y* is fuzzy *δ*-open in *X* iff for any *x*_*ε*_ ∈ *X* if *φ*(*x*_*ε*_)*qμ*, then *x*_*ε*_ *q*int_*δ*_(*φ* ^−1^(*μ*)).

**Proof**

Let *μ* ≤ *Y* be a fuzzy e-closed set and *φ*(*x*_*ε*_)*qμ*. Then, *x*_*ε*_ *qφ* ^−1^(*μ*) and, by hypothesis, *φ* ^−1^(*μ*) = int_*δ*_(*φ* ^−1^(*μ*)). From here, *x*_*ε*_ *q*int_*δ*_(*φ* ^−1^(*μ*)). The converse can be shown easily.

**Theorem 42 .**

For a fuzzy function *φ* : *X* → *Y*, if *φ*(*x*_*ε*_)*qμ*, for any fuzzy e-closed set *μ* ≤ *Y* and for any *x*_*ε*_ ∈ *X*, *x*_*ε*_ *q*int_*δ*_(*φ* ^−1^(*μ*)) iff there exists a fuzzy *δ*-open set *θ* such that *x*_*ε*_ *qθ* and *φ*(*θ*) ≤ *μ*.

**Proof**

Let *μ* ≤ *Y* be any fuzzy e-closed set and let *φ*(*x*_*ε*_)*qμ*. Then, *x*_*ε*_ *q*int_*δ*_(*φ* ^−1^(*μ*)). Take *θ* = int_*δ*_(*φ* ^−1^(*μ*)); then, *φ*(*θ*) = *φ*(int_*δ*_(*φ* ^−1^(*μ*))) ≤ *φ*(*φ* ^−1^(*μ*)) ≤ *μ*; *θ* is fuzzy *δ*-open in *X* and *x*_*ε*_ *qθ*.

Conversely, let *μ* ≤ *Y* be any fuzzy e-closed set and let *φ*(*x*_*ε*_)*qμ*. By hypothesis, there exists fuzzy *δ*-open set *θ* such that *x*_*ε*_ *qθ* and *φ*(*θ*) ≤ *μ*. This implies *θ* ≤ *φ* ^−1^(*μ*) and then *x*_*ε*_ *q*int_*δ*_(*φ* ^−1^(*μ*)).

**Theorem 43 .**

For a fuzzy function *φ* : *X* → *Y*, the following statements are equivalent:

(1) *f* is fuzzy completely *C*_*e*_-*I*(*rc*, *eo*).
(2) For every fuzzy e-closed set *μ* in *Y*, *φ* ^−1^(*μ*) is fuzzy regular open in *X*.
(3) For every fuzzy open set *μ*, *φ* ^−1^(*fe*-int(*μ*)) is fuzzy regular closed.
(4) For every fuzzy closed set *η*, *φ* ^−1^(*fe*-cl⁡(*η*)) is fuzzy regular open.
(5) For each *x*_*ε*_ ∈ *X* and each fuzzy e-closed set *μ* in *Y* containing *φ*(*x*_*ε*_), there exists a fuzzy regular open set *ρ* in *X* containing *x*_*ε*_ such that *φ*(*ρ*) ≤ *μ*.
(6) For each *x*_*ε*_ ∈ *X* and each fuzzy e-open set *μ* in *Y* non containing *φ*(*x*_*ε*_), there exists a fuzzy regular closed set *ν* in *X* noncontaining *x*_*ε*_ such that *φ* ^−1^(*μ*) ≤ *ν*.

**Proof**

(1) ⇔ (2): let *ρ* be a fuzzy e-open set in *Y*. Then, 1_*Y*_ − *ρ* is fuzzy e-closed. By (2), *φ* ^−1^(1_*Y*_ − *ρ*) = 1_*X*_ − *φ* ^−1^(*ρ*) is fuzzy regular open. Thus, *φ* ^−1^(*ρ*) is fuzzy regular closed. Thus, *φ* is fuzzy completely *C*_*e*_-*I*(rc, eo).The converse can be shown easily.

(1) ⇔ (3): let *μ* be a fuzzy open set. Since fe-int(*μ*) is fuzzy e-open, then by (1) it follows that *φ* ^−1^(fe-int(*μ*)) is fuzzy regular closed. The converse is easy to prove.

(2) ⇔ (4): let *η* be a fuzzy closed set. Since fe-cl(*η*) is fuzzy e-closed set, then by (2) it follows that *φ* ^−1^(fe-cl(*η*)) is fuzzy regular open. The converse is easy to prove.

(2) ⇔ (5): let *μ* be any fuzzy e-closed set in *Y* containing *φ*(*x*_*ε*_). By (2), *φ* ^−1^(*μ*) is fuzzy regular open set in *X* and *x*_*ε*_ ∈ *φ* ^−1^(*μ*). Take *ρ* = *φ* ^−1^(*μ*). Then, *φ*(*ρ*) ≤ *μ*. The converse can be shown easily.

(5) ⇔ (6): let *μ* be any fuzzy e-open set in *Y* noncontaining *φ*(*x*_*ε*_). Then, 1 − *μ* is a fuzzy e-closed set containing *φ*(*x*_*ε*_). By (5), there exists a fuzzy regular open set *ρ* in *X* containing *x*_*ε*_ such that *φ*(*ρ*) ≤ 1 − *μ*. Hence, *ρ* ≤ *φ* ^−1^(1 − *μ*) = 1 − *φ* ^−1^(*μ*) and *φ* ^−1^(*μ*) ≤ 1 − *ρ*. Take *ν* = 1 − *ρ*. We obtain that *ν* is a fuzzy regular closed set in *X* noncontaining *x*_*ε*_. The converse can be shown easily.

**Theorem 44 .**

Let *φ*_1_ : *X* → *Y* be a function and let *φ*_2_ : *X* → *X* × *Y* be the fuzzy graph function of *φ*_1_, defined by *φ*_2_(*x*_*ε*_) = (*x*_*ε*_, *φ*_1_(*x*_*ε*_)) for every *x*_*ε*_ ∈ *X*. If *φ*_2_ is fuzzy completely *C*_*e*_-*I*(*rc*, *eo*), then *φ*_1_ is fuzzy completely *C*_*e*_-*I*(*rc*, *eo*).

**Proof**

Let *μ* be a fuzzy e-closed set in *Y*; then, 1_*X*_ × *μ* is a fuzzy e-closed set in *X* × *Y*. Since *φ*_2_ is fuzzy completely *C*_*e*_-*I*(rc, eo), then *φ*_1_^−1^(*μ*) = *φ*_2_^−1^(1_*X*_ × *μ*) is fuzzy regular open in *X*. Thus, *φ*_1_ is fuzzy completely *C*_*e*_-*I*(rc, eo).

**Theorem 45 .**

The following holds for functions *φ*_1_ : *X* → *Y* and *φ*_2_ : *Y* → *Z*:

(a) If *φ*_1_ : *X* → *Y* is fuzzy *C*_*e*_-*I*(*ec*, *eo*) and *φ*_2_ : *Y* → *Z* is fuzzy completely *C*_*e*_-*I*(*rc*, *eo*), then *φ*_2_∘*φ*_1_ : *X* → *Z* is fuzzy e-irresolute.
(b) If *φ*_1_ : *X* → *Y* is fuzzy completely *C*_*e*_-*I*(*rc*, *eo*) and *φ*_2_ : *Y* → *Z* is fuzzy *C*_*e*_-continuous, then *φ*_2_∘*φ*_1_ : *X* → *Z* is fuzzy completely continuous.

**Definition 46 .**

A fuzzy filter base *ξ* is said to be fuzzy e-convergent to a fuzzy point *x*_*ε*_ in *X* if for any fuzzy e-open set *η* in *X* containing *x*_*ε*_ there exists a fuzzy set *ρ* ∈ *ξ* such that *ρ* ≤ *η*.

**Theorem 47 .**

If a fuzzy function *φ* : *X* → *Y* is fuzzy completely *C*_*e*_-*I*(*rc*, *eo*) for each fuzzy point *x*_*ε*_ ∈ *X* and each fuzzy filter base *ξ* in *X* is fuzzy rc-convergent to *x*_*ε*_, then the fuzzy filter base *φ*(*ξ*) is fuzzy e-convergent to *φ*(*x*_*ε*_).

**Proof**

Let *x*_*ε*_ ∈ *X* and let *ξ* be any fuzzy filter base in *X* which is fuzzy rc-converging to *x*_*ε*_. Since *φ* is fuzzy completely *C*_*e*_-*I*(rc, eo), then for any fuzzy e-open set *η* in *Y* containing *φ*(*x*_*ε*_), there exists a fuzzy regular closed set *ρ* in *X* containing *x*_*ε*_ such that *φ*(*ρ*) ≤ *η*. Since *ξ* is fuzzy rc-converging to *x*_*ε*_, there exists a *δ* ∈ *ξ* such that *δ* ≤ *ρ*. This means that *φ*(*δ*) ≤ *ρ* and therefore the fuzzy filter base *φ*(*ξ*) is fuzzy e-convergent to *φ*(*x*_*ε*_).

**Theorem 48 .**

If *φ* : *X* → *Y* is a fuzzy completely *C*_*e*_-*I*(*rc*, *eo*) surjection and *X* is fuzzy S-closed, then *Y* is fuzzy e-compact.

**Proof**

Suppose that *φ* : *X* → *Y* is a fuzzy completely *C*_*e*_-*I*(rc, eo) surjection and *X* is fuzzy S-closed. Let {*ν*_*i*_}_*i*∈*I*_ be a fuzzy e-open cover of *Y*. Since *φ* is a fuzzy completely *C*_*e*_-*I*(rc, eo), then {*φ* ^−1^(*ν*_*i*_)}_*i*∈*I*_ is fuzzy regular closed cover of *X* and hence there exists finite set *I*_0_ of *I* such that *X* = ⋁{*φ* ^−1^(*ν*_*i*_); *i* ∈ *I*_0_}. Therefore, we have *Y* = ⋁{*ν*_*i*_; *i* ∈ *I*_0_} and *Y* is fuzzy e-compact.

**Theorem 49 .**

If *φ* : *X* → *Y* is a fuzzy completely *C*_*e*_-*I*(*rc*, *eo*) injection and *Y* is fuzzy *e*-*T*_1_, then *X* is fuzzy weakly Hausdorff.

**Proof**

Suppose *Y* is fuzzy *e*-*T*_1_. For any distinct fuzzy points *x*_*ε*_ and *y*_*υ*_ in *X*, there exist fuzzy e-open sets *η* and *ρ* in *Y*. Since *φ* is injective, *φ*(*x*_*ε*_) ∈ *η*, *φ*(*y*_*υ*_) ∉ *η*, *φ*(*x*_*ε*_) ∉ *ρ*, and *φ*(*y*_*υ*_) ∈ *ρ*. Since *φ* is fuzzy completely *C*_*e*_-*I*(rc, eo), *φ* ^−1^(*η*) and *φ* ^−1^(*ρ*) are fuzzy regular closed sets of *X* such that *x*_*ε*_ ∈ *φ* ^−1^(*η*), *y*_*υ*_ ∉ *φ* ^−1^(*η*), *x*_*ε*_ ∉ *φ* ^−1^(*ρ*), and *y*_*υ*_ ∈ *φ* ^−1^(*ρ*). This shows that *X* is fuzzy weakly Hausdorff.

**Theorem 50 .**

If *φ* : *X* → *Y* is a fuzzy completely *C*_*e*_-*I*(*rc*, *eo*) injection and *Y* is fuzzy e-normal, then *X* is fuzzy strongly normal.

**Proof**

Let *η* and *ρ* be disjoint nonempty fuzzy closed sets of *X*. Since *φ* is injective, *φ*(*η*) and *φ*(*ρ*) are disjoint fuzzy closed sets. Since *Y* is fuzzy e-normal, there exist fuzzy e-open sets *μ* and *λ* such that *φ*(*η*) ≤ *μ* and *φ*(*ρ*) ≤ *λ* and *μ*∧*λ* = 0. This implies that fe-cl(*μ*) and fe-cl(*λ*) are fuzzy e-closed sets in *Y*. Then, since *φ* is fuzzy completely *C*_*e*_-*I*(rc, eo), *φ* ^−1^(fe-cl(*μ*)) and *φ* ^−1^(fe-cl(*λ*)) are fuzzy regular open sets. Then, *η* ≤ *φ* ^−1^(fe-cl(*μ*)) and *ρ* ≤ *φ* ^−1^(fe-cl(*λ*)) and *φ* ^−1^(fe-cl(*μ*)) and *φ* ^−1^(fe-cl(*λ*)) are disjoint; by definition *X* is fuzzy strongly normal.

**Definition 51 .**

A fuzzy topological space (*X*, *τ*) is said to be fuzzy *e*-*T*_0_(*r*-*T*_0_ [14]) if for every fuzzy set *λ* of *X* can be written in the form *λ* = ⋁_*i*∈*I*_⋀_*j*∈*J*_ *λ*_*ij*_, where *λ*_*ij*_ are fuzzy e-open (fuzzy regular open) or fuzzy e-closed (fuzzy regular closed) sets of *Y*.

**Theorem 52 .**

If *φ* : *X* → *Y* is a fuzzy completely *C*_*e*_-*I*(*rc*, *eo*) injection and *Y* is fuzzy *e*-*T*_0_, then *X* is fuzzy *r*-*T*_0_.

**Proof**

Let *η* be a any fuzzy set of *X*. Since *Y* is fuzzy *e*-*T*_0_, *φ*(*η*) is fuzzy e-open set of *Y*. Then, *φ*(*η*) = ⋁_*i*∈*I*_⋀_*j*∈*J*_ *λ*_*ij*_, where *λ*_*ij*_ are fuzzy e-open set or fuzzy e-closed sets of *Y*. Since *φ* is completely *C*_*e*_-*I*(rc, eo) injection we have *η* = *φ* ^−1^(*φ*(*η*)) = *φ* ^−1^(⋁_*i*∈*I*_⋀_*j*∈*J*_ *λ*_*ij*_) = ⋁_*i*∈*I*_⋀_*j*∈*J*_ *φ* ^−1^(*λ*_*ij*_), where *φ* ^−1^(*λ*_*ij*_) are fuzzy regular open sets or fuzzy regular closed sets of *X*. Thus, *X* is fuzzy *r* − *T*_0_.

**Theorem 53 .**

If *φ* : *X* → *Y* is a fuzzy completely *C*_*e*_-*I*(*rc*, *eo*) injection and *Y* is fuzzy *e*-*T*_2_, then *X* is fuzzy Urysohn.

**Proof**

Let *x*_*ε*_ and *y*_*υ*_ be any two distinct fuzzy points in *X*. Since *φ* is injective, *φ*(*x*_*ε*_) ≠ *φ*(*y*_*υ*_) in *Y*. Since *Y* is fuzzy *e*-*T*_2_, there exist fuzzy e-open sets *η* and *ρ* in *Y* such that *φ*(*x*_*ε*_) ∈ *η* and *φ*(*y*_*υ*_) ∈ *ρ* and *η*∧*ρ* = 0. This implies that fe-cl(*η*) and fe-cl(*ρ*) are fuzzy e-closed sets in *Y*. Then, since *φ* is fuzzy completely *C*_*e*_-*I*(rc, eo), there exists fuzzy regular open sets *δ* and *γ* in *X* containing *x*_*ε*_ and *y*_*υ*_, respectively, such that *φ*(*δ*) ≤ fe-cl(*η*) and *φ*(*γ*) ≤ fe-cl(*ρ*). This implies that *δ* ≤ *φ* ^−1^(fe-cl(*η*)) and *γ* ≤ *φ* ^−1^(fe-cl(*ρ*)); we have that *φ* ^−1^(fe-cl(*η*)) and *φ* ^−1^(fe-cl(*ρ*)) are disjoint and hence cl(*δ*)∧cl(*γ*) = 0; by definition, *X* is fuzzy Urysohn.
